# Sequencing and analysis of globally obtained human parainfluenza viruses 1 and 3 genomes

**DOI:** 10.1371/journal.pone.0220057

**Published:** 2019-07-18

**Authors:** Michael E. Bose, Susmita Shrivastava, Jie He, Martha I. Nelson, Jayati Bera, Nadia Fedorova, Rebecca Halpin, Christopher D. Town, Hernan A. Lorenzi, Paolo Amedeo, Neha Gupta, Daniel E. Noyola, Cristina Videla, Tuckweng Kok, Amelia Buys, Marietjie Venter, Astrid Vabret, Samuel Cordey, Kelly J. Henrickson

**Affiliations:** 1 Midwest Respiratory Virus Program, Medical College of Wisconsin, Milwaukee, WI, United States of America; 2 J. Craig Venter Institute, Rockville, MD, United States of America; 3 Fogarty International Center, National Institutes of Health, Bethesda, MD, United States of America; 4 Departamento de Microbiología, Facultad de Medicina, Universidad Autónoma de San Luis Potosí, San Luis Potosí, Mexico; 5 Clinical Virology Laboratory, Centro de Educación Médica e Investigaciones Clínicas (CEMIC) University Hospital, Buenos Aires, Argentina; 6 School of Molecular and Biomedical Science, University of Adelaide, Adelaide, Australia; 7 Centre for Respiratory Diseases and Meningitis, National Institute for Communicable Diseases, Sandringham, South Africa; 8 Zoonotic, arbo and respiratory virus program, Department Medical Virology, University of Pretoria, Pretoria, South Africa; 9 Normandie Université, Caen, France; 10 Groupe de Recherche sur l'Adaptation Microbienne (GRAM), Université de Caen, Caen, France; 11 Laboratoire de Virologie, Centre Hospitalier Universitaire de Caen, Caen, France; 12 Division of Infectious Diseases and Laboratory of Virology, University of Geneva Hospitals, Geneva, Switzerland; Defense Threat Reduction Agency, UNITED STATES

## Abstract

Human Parainfluenza viruses (HPIV) type 1 and 3 are important causes of respiratory tract infections in young children globally. HPIV infections do not confer complete protective immunity so reinfections occur throughout life. Since no effective vaccine is available for the two virus subtypes, comprehensive understanding of HPIV-1 and HPIV-3 genetic and epidemic features is important for diagnosis, prevention, and treatment of HPIV-1 and HPIV-3 infections. Relatively few whole genome sequences are available for both HPIV-1 and HPIV-3 viruses, so our study sought to provide whole genome sequences from multiple countries to further the understanding of the global diversity of HPIV at a whole-genome level. We collected HPIV-1 and HPIV-3 samples and isolates from Argentina, Australia, France, Mexico, South Africa, Switzerland, and USA from the years 2003–2011 and sequenced the genomes of 40 HPIV-1 and 75 HPIV-3 viruses with Sanger and next-generation sequencing with the Ion Torrent, Illumina, and 454 platforms. Phylogenetic analysis showed that the HPIV-1 genome is evolving at an estimated rate of 4.97 × 10^−4^ mutations/site/year (95% highest posterior density 4.55 × 10^−4^ to 5.38 × 10^−4^) and the HPIV-3 genome is evolving at a similar rate (3.59 × 10^−4^ mutations/site/year, 95% highest posterior density 3.26 × 10^−4^ to 3.94 × 10^−4^). There were multiple genetically distinct lineages of both HPIV-1 and 3 circulating on a global scale. Further surveillance and whole-genome sequencing are greatly needed to better understand the spatial dynamics of these important respiratory viruses in humans.

## Introduction

Human Parainfluenza viruses (HPIV) belong to the Paramyxovirus family and are important causes of respiratory tract infections in young children, elderly individuals, and the immunocompromised globally. Serologic evidence shows that most children over five years of age have been infected by HPIV [1]. There are four types of HPIV with HPIV-1 and 3 belonging to the Human respirovirus 1 and 3 species of the Respirovirus genus and HPIV-2 and 4 belonging to the Human rubulavirus 2 and 4 species of the Rubulavirus genus. HPIV-1 and 3 cause more illness than the other two types and are therefore more studied. These two types have been reported to have distinct clinical and epidemiologic features. HPIV-1 is the most common pathogen associated with croup while HPIV-3 ranks only behind respiratory syncytial viruses (RSV) as a cause of bronchiolitis and viral pneumonia among infants and young children [2–4]. In children less than 5 years of age it has been estimated that HPIV-1 causes 0.3–1.6 hospitalizations per 1000 children and HPIV-3 causes 0.5–2.6 hospitalizations per 1000 children in the United States [3–5]. Both HPIV-1 and 3 also cause serious lower respiratory tract disease and death in the immunocompromised and elderly. HPIV-1 infections have been epidemic in the fall of odd-numbered years since 1973 and HPIV-3 infections are usually widespread in the United States during late spring and summer [3,6]. HPIV infections do not confer complete protective immunity and individuals get recurrent infections throughout their life. No effective vaccine is yet available for these viruses and further studies are needed to better understand the disease burden of these viruses [2]. A better understanding of HPIV-1 and 3 genome diversity, evolution, and epidemiological features is key for the development of improved diagnostics tools, prevention strategies, and treatments for HPIV-1 and 3 infections. However, major progress in this field has been hampered in part by the limited amount of HPIV-1 and 3 whole genome sequence data available to the scientific community in public repositories. To contribute to filling this gap in knowledge, our study sought to carry out a large-scale whole genome sequencing and analysis of HPIV-1 and 3 strains collected from multiple countries and years. HPIV-1 and 3 samples were collected from Argentina, Australia, France, Mexico, South Africa, Switzerland, and the USA during the period 2003–2011 and genome sequenced using a combination of Sanger and Next-Generation Sequencing (NGS) technologies (Ion Torrent, Illumina, and 454 platforms). Here, we present the results of a comparative analysis of 40 HPIV-1 and 75 HPIV-3 novel genome sequences, in combination with additional HPIV sequences available in Genbank. Our findings provide further insights into HPIV1 and HPIV3 worldwide genome diversity and evolution.

## Materials and methods

### Ethics statement

Samples from the Virology Laboratory of CEMIC, University of Geneva Hospitals, National Reference Center for Measles and Paramyxoviridae, and University of Adelaide—Institute of Medical & Veterinary Science Adelaide were collected for routine viral diagnostic testing and de-identified prior to shipment to the Medical College of Wisconsin. Samples from the Autonomous University of San Luis Potosí were collected with written informed consent as approved by the Research and Ethics Committee. Characterization of strains by sequencing was approved and monitored by the human ethics committee, University of Pretoria (25/2006), and ethical approval for surveillance and virus characterization at National Institute for Communicable Diseases was obtained from the Human Research Ethics Committee (Medical), University of the Witwatersrand, Johannesburg. At Children’s Hospital of Wisconsin and Froedtert Hospital samples were collected with written informed consent as approved by the Children’s Hospital of Wisconsin Human Research Review Board. For samples collected from minors written informed consent was obtained from their parent or guardian on their behalf. Some samples had been de-identified and where tested under a protocol approved by the Children’s Hospital of Wisconsin Human Research Review Board.

### Sample collection and HPIV identification from various locations

Samples were collected retrospectively as part of a larger paramyxovirus sequencing project which also included RSV and human metapneumovirus. The RSV sequencing part of the project was published previously [7]. There was some overlap in locations that provided RSV and HPIV samples. Even though collection details for some of these locations has already been published previously, they are repeated here for clarity.

#### The Autonomous University of San Luis Potosí (San Luis Potosí, Mexico)

Respiratory samples were collected from pediatric subjects as part of research projects carried out to analyze the epidemiology of viral respiratory infections and as part of a hospital-based infection control program [8–11]. The research projects were approved by the corresponding Research and Ethics Committees. Samples were obtained by nasal wash or pharyngeal swab, and they were processed as described in Noyola et al. (2004) [8]. Viral testing was carried out directly on respiratory samples. HPIV identification was performed with a direct fluorescent antibody assay using the Respiratory DFA viral screening and identification kit (Light Diagnostics, Chemicon International, Temecula, CA, subsequently Light Diagnostics, Millipore Corporation, Temecula, CA) which detects seven respiratory viruses including adenovirus, influenza A, influenza B, HPIV-1, HPIV-2, HPIV-3, and RSV. After viral detection, respiratory samples were stored at -70°C until they were shipped on dry ice for sequence analysis.

#### Virology laboratory of CEMIC (Buenos Aires, Argentina)

Respiratory samples from hospitalized pediatric patients less than 1 year of age were submitted for routine viral diagnostics. Nasopharyngeal aspirates or swabs were obtained in a transport media (Hanks plus 2% FCS, penicillin, streptomycin, and amphotericin B). Samples were processed for antigen detection on pelleted cells by indirect immunofluorescence with monoclonal antibodies against RSV, adenovirus, influenza A and B and parainfluenza (EMD Millipore, Billerica, MA, USA) [12,13]. A fluorescein labeled anti-mouse IgG was used (Sigma-Aldrich Corp., St. Louis, MO, USA). Readings were performed with a C. Zeiss microscope provided with epifluorescent equipment and a mercury lamp. An aliquot of the sample was preserved in liquid nitrogen.

#### University of Adelaide—Institute of Medical & Veterinary Science (Adelaide, Australia)

Nasal pharyngeal aspirates from pediatric patients less than 15 years of age were submitted for routine viral diagnostics and tested within eight hours of collection for seven viruses (adenovirus, influenza A and B, HPIV 1, 2, and 3 and RSV) and *Mycoplasma pneumoniae* [14]. The viruses and *M*. *pneumoniae* were identified directly from the samples by specific antibodies using in-house developed enzyme immunoassays. Samples were also inoculated into 96-well microwell cell cultures, spun at 1000 × g for 1hr at 35°C and incubated for 5–6 days at 37°C prior to testing the inoculated cell culture fluids for viruses by enzyme immunoassay [15]. Following testing the remaining volumes were then stored at 4°C for less than three days and then stored at -70°C long term. The samples used in this report were randomly selected without any specific patient identifications.

#### Department of Medical Virology, University of Pretoria (Pretoria, South Africa) and National Institute for Communicable Diseases (NICD) (Johannesburg, South Africa)

Respiratory samples from 2007–2010 were from pediatric and adult patients and submitted either as part of outpatients enrolled in the NICD Viral Watch programme [16] or for routine diagnosis from patients with lower respiratory tract infection, hospitalized in the Kalafong and Steve Biko Academic hospitals. Testing in 2007 was performed at the Tshwane National Health Laboratory Service laboratory, Department Medical Virology, University of Pretoria by direct immunofluorescent assay followed by confirmation by RT-PCR, as described previously [17]. At NICD, samples from 2008–2009 were tested in shell vials and direct immunofluorescence [18], from 2010 onwards samples were tested with RT-PCR [19]. Some viruses were amplified in tissue culture for this study. Samples were stored at -80°C.

#### National Reference Center for Measles and Paramyxoviridae, Caen University Hospital (Caen, France)

Respiratory samples from pediatric and adult patients were submitted for routine diagnostics for 15 respiratory viruses (adenovirus, influenza A and B, HPIV 1, 2, 3, and 4, RSV A and B, human metapneumovirus, human coronaviruses 229E, OC43, NL63 and HKU1, and bocaviruses) and four intracellular bacteria including *Mycoplasma pneumoniae*. These viral and bacterial targets were detected directly from the samples using the RespiFinder assay (PathoFinder B.V., Maastricht, Netherlands). Samples were divided in aliquots and stored frozen at -80°C.

#### Laboratory of Virology, University of Geneva Hospitals (Geneva, Switzerland)

Nasopharyngeal swabs and aspirates were collected from pediatric and adult patients for routine viral diagnostics for a panel of respiratory viruses (adenovirus, influenza A and B, metapneumovirus, HPIV 1, 2, and 3, rhinovirus, enterovirus and RSV). HPIV were identified by a published in house real-time RT-PCR assay [20]. Samples were stored at -80°C.

#### The Midwest Respiratory Virus Program, Children’s Hospital of Wisconsin, and Dynacare Laboratories (Milwaukee, Wisconsin, USA)

Nasopharyngeal swab samples were collected from pediatric and adult patients with informed consent under an IRB protocol or through routine clinical diagnosis and de-identified. HPIVs were identified by an in-house real-time RT-PCR. Sample remnants were kept at -80°C.

Samples from all locations were frozen at -70°C or less and held frozen until shipped on dry ice to the Midwest Respiratory Virus Program. Once received they were stored at -80°C until further processing.

### Sequencing HPIV genomes

For samples sequenced using the Sanger technology, genome specific PCR primers tiling across the entire genome were designed from the respective HPIV-1 and HPIV-3 consensus sequences using an automated PCR primer design pipeline developed at the J. Craig Venter Institute (JCVI) with an average amplicon length of 650 bp and an overlap of 150 bp [21]. Each primer had an M13 sequence tag at the 5’ end used for Sanger sequencing. They were then pruned to a minimal set of 95 primers with at least three-fold amplicon coverage at every base position of the consensus. The HPIV-1 primer sequences (S1 Text) were generated based on a consensus sequence using the Genbank accessions NC_003461, JQ901990, JQ901989, JQ901975 and JQ901972; while the HPIV-3 primers (S2 Text) were generated using a consensus sequence generated from the accessions: AB012132, Z11575, EU424062, FJ455842 and EU326526.

Viral RNA was extracted from 100–400 μl of the samples using the NucliSENS easyMag (bioMerieux, Inc., Durham, NC, USA) with elution in 25 μl at the Midwest Respiratory Virus Program. After extraction the RNA was shipped on dry ice to the JCVI. There, RNA from each sample was subjected to RT-PCR using a Qiagen One-step RT-PCR kit (Qiagen, Hilden, Germany) to produce 650 bp amplicons from each of the 95 primer pairs for both HPIV-1 and HPIV-3 samples. PCR products were treated with a mixture of exonuclease and shrimp alkaline phosphatase to remove primers and dNTPs, and then Sanger sequencing was performed with M13 primers.

For samples sequenced using Ion Torrent, 100ng of pooled DNA amplicons were sequenced as described previously [22].

For samples sequenced using SISPA and 454/Illumina, 100ng of pooled DNA amplicons were randomly amplified and prepared for next-generation sequencing using a sequence independent single primer amplification (SISPA) method as initially described by Djikeng et al. [23]. Briefly, 100ng of amplified viral DNA were denatured in the presence of 6% w/v DMSO and a chimeric oligonucleotide containing a known barcode 22nt sequence followed by a 3’ random hexamer. A Klenow reaction was prepared with the denatured DNA template by adding NEB buffer II, 3’-5’ exo- Klenow (New England Biolabs, Ipswich, MA, USA), and dNTPs (Thermo Fisher Scientific, Waltham, MA, USA). The Klenow reaction was incubated at 37°C for 60 min, followed by incubation at 75°C for 10 min. The resulting cDNA was randomly amplified by PCR using Promega GoTaq Hot Start Polymerase (Promega Corporation, Madison, WI, USA) at 35 cycles (denaturation: 30 sec, 94°C; annealing: 30sec, 55°C; extension: 48 sec, 68°C). PCR reactions contained primers corresponding to the known 22nt barcode sequence from the oligonucleotide utilized in the previous Klenow step. The resulting cDNA was then treated with Exonuclease I at 37°C for 60 min, followed by incubation at 72°C for 15 min.

SISPA products were normalized and pooled into a single reaction that was purified using the QIAquick PCR purification kit (Qiagen, Hilden, Germany). Sample was further gel purified to select for SISPA products 300-500bp in size for Illumina sequencing and 500-800bp in size for 454 sequencing. Two aliquots were then submitted for sequencing with 454 and Illumina sequencing technologies. 454 sequencing was performed on the GS FLX+. A rapid library was created, and samples were sequenced on one half plate. Illumina sequencing was performed on the Illumina MiSeq v2 with 250bp paired-end reads.

### Assembly and annotation

For samples sequenced with Sanger, sequencing reads were trimmed to eliminate amplicon primer and low-quality sequences. They were then assembled with CLC using the *clc_ref_assemble_long* program [24]. Draft assemblies were evaluated, and targeted PCR-based sequencing reactions were conducted to close gaps and improve sequence coverage.

For samples sequenced with a combination of Sanger, 454, Illumina, and Ion Torrent, the samples were assembled, validated, and annotated as described previously with minimal modification [22]. After the sequences had been assembled using CLC Bio’s *clc_novo_assemble* program [25], the resulting contigs were searched against custom full-length HPIV-1 or HPIV-3 nucleotide databases to find the closest reference sequence which was then used for mapping the sequence.

All sequences generated as part of this study were submitted to Genbank as part of the Bioproject id PRJNA73053 for HPIV-1 with accessions KF530195 –KF530224 and KF687307 –KF687316 and PRJNA73055 for HPIV-3 with accessions KF530225 –KF530257 and KF687317 –KF687358.

### Recombinant analysis

All HPIV-1 and 3 genome sequences with collection years available in Genbank as of 3/7/2019 were downloaded and combined with the genomes produced in this study. The genomes were aligned using MAFFT [26]. Any sequence that did not contain all of the complete coding sequences (CDS) for all of the HPIV genes was deleted from the alignment. Recombination was predicted for each alignment using the RDP, GENECONV, Chimaera, MaxChi, BootScan, SiScan, and 3Seq algorithms in RDP4.8 [27]. Recombination events were only considered to be potentially real if they were predicted by at least three of the algorithms. Any potential recombinants were deleted from the alignments used for subsequent analyses. One other sequence (MH678676) was removed due to a significant number of unresolved positions and another (KY234287) for containing a large insertion that could not be verified.

### Entropy plots

To assess the degree of variability of the HPIV-1 and 3 proteomes, the complete genome alignments were trimmed to contain only the protein coding sequences for the N, P, M, F, HN, and L genes and then translated into predicted protein sequences. The entropy values for each amino acid position were calculated using the Entropy (H(x)) plot function in BioEdit 7.0. The entropy data was imported into Microsoft Excel where the number of positions with variants in more than one sequence were counted, the percent of variable positions were determined for each protein, and the data was plotted.

### Positive selection

The same set of sequences used for the recombinant analysis was split into the individual CDS for the N, P, M, F, HN, and L genes. All additional complete CDS with collection years were retrieved from Genbank and added to these sets of sequences. Sequences were aligned again with MAFFT. Pervasive diversifying selection was determined for each alignment using the SLAC, FEL, and FUBAR algorithms available on the Datamonkey webserver [28–31]. Additionally, episodic diversifying selection was checked with the MEME algorithm [30]. For the HN CDS of HPIV3 the number of sequences had to be reduced to less than 500 for SLAC, FEL, and FUBAR and less than 400 for MEME to run successfully. Identical nucleotide sequences and some with nucleotide substitutions that caused no amino acid changes were removed to reach these numbers. Sites were only considered positive if they met the cutoffs for two or more of the algorithms, which were a p-value of less than 0.05 for SLAC, FEL, and MEME and Bayes factor/posterior probability of greater than 0.95 for FUBAR.

### Phylogenetic analyses

The whole genome sequence and complete CDS alignments were analyzed with the Bayesian method of phylogenetic inference in BEAST v1.8.2 [32].BEAUti v1.8.0 was used to generated the xml files for use in BEAST with the collection dates used to assign tip dates with variable precision. The HKY model of nucleotide substitution was used with the gamma + invariants site model with four gamma categories, strict clock model, and the piecewise-constant Bayesian Skyline tree prior with 10 groups. For the complete CDS analyses sites were partitioned into 3 codon positions. The full genome and CDS sequences were run on 100 million iterations for HPIV-1. For HPIV-3 more iterations were needed to reach appropriate effective sample size values so two to five replicate runs of 100 million were performed. Results were analyzed with Tracer v1.6 to find the evolutionary rates [33]. Summary trees were produced using TreeAnnotator v1.8.0 to infer maximum likelihood trees for the genome and full CDS. The resulting trees were visualized and annotated with FigTree v1.4.0.

## Results and discussion

As part of this study, 40 HPIV-1 and 75 HPIV-3 genomes were sequenced in total. Of these, 21 HPIV-1 and 51 HPIV-3 genomes were high quality sequences resulting in complete whole genome assemblies. The 43 remaining assemblies contained at least one sequence gap, either due to low quality sequence at specific regions or insufficient sequencing data, resulting in more than one contig per genome assembly. Ten HPIV-1 and 42 HPIV-3 genomes were sequenced using Sanger, while the remaining samples were sequenced using NGS platforms. One HPIV-3 sample was sequenced completely with both Sanger and NGS methods. Information on the sequenced viruses can be found in S1 Table and S2 Table.

### Recombination

No recombination events were predicted in the HPIV-1 genomes. In the HPIV-3 genomes there were five genomes that were predicted to be recombinant. Two of these genomes were produced in this study (KF530247 and KF530229). Upon further inspection of the reads for these samples it became clear that they were a mix of reads from more than one virus with the percentage of reads representing each virus varying greatly across the genome. A deep sequencing analysis on KF530247 and KF530229 showed the presence of a mix of major and minor variants at multiple positions across the genome with high confidence. We could not tease apart the major and minor variants from the samples and therefore, for samples KF530247 and KF530229 the consensus sequence represented just the major variant. The amount of minor variant at positions across the genome were as much as 48%. Sample KF530247 had sites with minor frequency variants above 10% at 26 positions and sample KF530229 had minor variants above 10% at 11 positions across the genome. There were many other sites in both genomes where the frequency of minor variants was less than 10%. The other three potential HPIV-3 recombinant genomes (FJ455842, MH678681, and EU326526) were published by other research groups and lacked the sequencing reads required to confirm they were true recombinants. The publishers of FJ455842 argued that this virus was an example of natural recombination in HPIV-3 [34]. However, the only evidence shown is from recombination prediction algorithms, and the putative breakpoint falls in the overlap region of two amplicons used for sequencing. In our experience recombination events like this are typically the result of two different viruses being amplified and sequenced. Appropriate evidence for the recombination event would be individual reads spanning the breakpoint showing sequence that matches one parent on each side.

### Entropy plots of the HPIV-1 and 3 protein sequences

**Fig 1:**
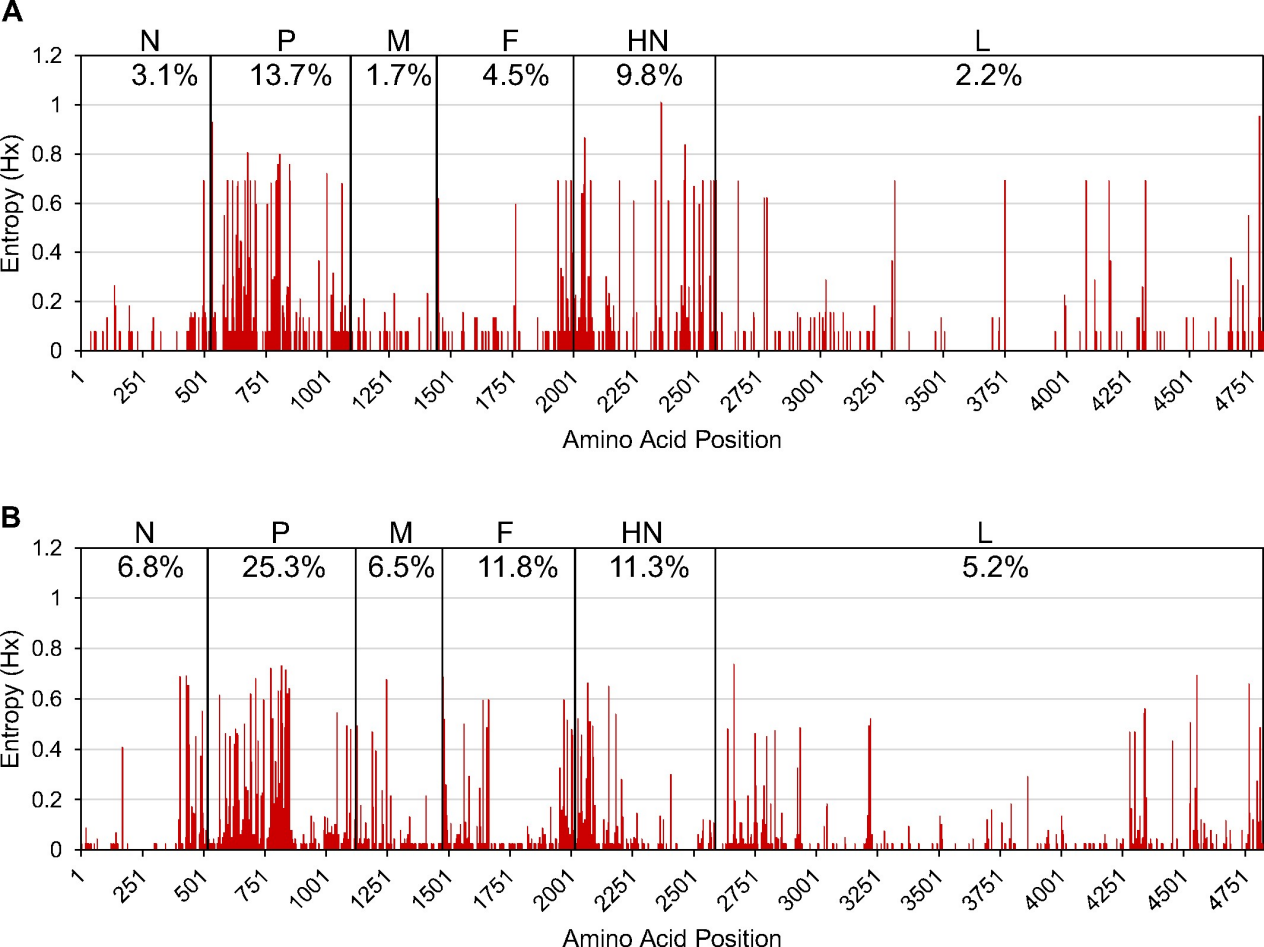
**Entropy plots of concatenated HPIV-1 (A) and HPIV-3 (B) protein sequences.** For each amino acid position in the protein sequence, higher entropy values represent greater amino acid variation. Abbreviated protein sequence names are shown across the top of the plot in the order in which coding sequences are arranged in the genome. The percentage values show the percentage of positions in the protein that have a mutation present in more than one sequence.

Interestingly, our analysis revealed that all of the proteins of HPIV-3 harbored more variable sites than the equivalent proteins in the HPIV-1 virus (Fig 1). The increased percentage of variable sites may at least partially be due to there being four times more sequences for HPIV-3 allowing for a larger amount of the population’s variation to be observed. From these plots it was clear that the P protein was the most variable in both HPIV-1 and 3. The P protein is important for RNA transcription and genome replication. A region (78–320) of the P protein that was previously identified as not being necessary for RNA synthesis in Sendai virus (a close relative of HPIV-1) contained 66% of the variable positions in the HPIV-1 P protein, even though the 243 residues represent only 43% of the protein sequence [35]. This protein is not well conserved between HPIV-1 and 3 so it is not clear exactly how the functional regions line up position-wise, but a similar region in HPIV-3 contained 55% of the variable positions. Perhaps since this region is not critical for the primary functions of the protein it is more accommodating of mutations. Often the HN gene is selected as the target gene to analyze the evolution of HPIVs because of its relatively high variability [36,37]. Our results suggest that the P gene could also be useful as a molecular marker for evolutionary analyses and epidemiology research. The entropy analysis also highlights more conserved regions of the genome that could be useful for the development of robust diagnostics and potential vaccines.

### Positively selected sites

We used the SLAC, FEL, MEME, and FUBAR algorithms on the Datamonkey webserver to identify potential positively selected sites for each of the coding regions in HPIV-1 and 3 (Table 1). The positively selected site at codon 5 of the HPIV-1 F CDS is a part of the hydrophobic signal peptide, which is important for insertion of the premature fusion peptide into the rough endoplasmic reticulum [38]. Later in the fusion maturation process the signal peptide is cleaved from the protein. Since the signal peptide is not present in the mature fusion protein or virus and therefore should not be exposed to the host immune response it is unclear what could be the potential source of the positive/diversifying selective pressure on this site. In HPIV-3, site 108 of the F protein was found to be under positive selection. This site is part of the cleavage sequence which has historically been described as amino acids 106–109 with the motif R-T-K-R but in modern sequences is more commonly R-T-E-R (present in >95% of sequences). There are seven sequences that have a G at this position and one that has an R. It is interesting that there are positively charged (K and R), negatively charged (E), and neutral (G) amino acids at this position. R and E have previously been observed in this position and were found to have no effect on replication in tissue culture or the respiratory tract of rhesus monkeys [39]. Between HPIV-1 and 3 there were six sites predicted to be under positive selection in the P protein. All of these sites fall in the region we described above that is highly variably but has been shown to not be essential for the function of the protein in Sendai virus. The two sites under positive selection in the HPIV-3 HN protein appear to frequently switch between two functionally similar amino acids with site 58 being S or N and site 168 being K or R. Both sites require only a single nucleotide change to change between the two amino acids. Site 440 of the HPIV-3 N protein is a Q in most sequences, but in multiple independent instances has switched to an R. The MEME algorithm predicted that there were 21 codons across the genome affected by episodic selection for HPIV-1 and 14 codons for HPIV-3 (S3 Table).

**Table 1:** Sites under positive selection for HPIV-1 and HPIV-3.

Virus	Gene	Codon	SLAC p-value	FEL p-value	FUBAR Post. Pr.	MEME p-value
HPIV-1	P	159	0.16	**0.029**^**a**^	**0.98**	**0.0002**
	F	5	0.23	**0.036**	**0.973**	**0.0448**
HPIV-3	N	440	**0.0046**	**0.002**	**1**	**0.0038**
	P	109	**0.026**	**0.008**	**0.961**	**0.0135**
	P	142	0.0976	**0.01**	**0.955**	**0.0166**
	P	196	0.0967	**0.013**	**0.962**	**0.0213**
	P	258	**0.0484**	0.072	**0.968**	0.0947
	P	302	0.156	**0.012**	**0.964**	**0.0016**
	F	108	**0.0244**	**0.032**	**0.982**	**0.0475**
	HN	58	**0.0342**	**0.019**	**0.974**	**0.0289**
	HN	168	**0.0459**	**0.027**	0.938	**0.0394**

^a^ Bold font indicates values that meet the algorithm specific threshold.

### Evolutionary rates

**Fig 2:**
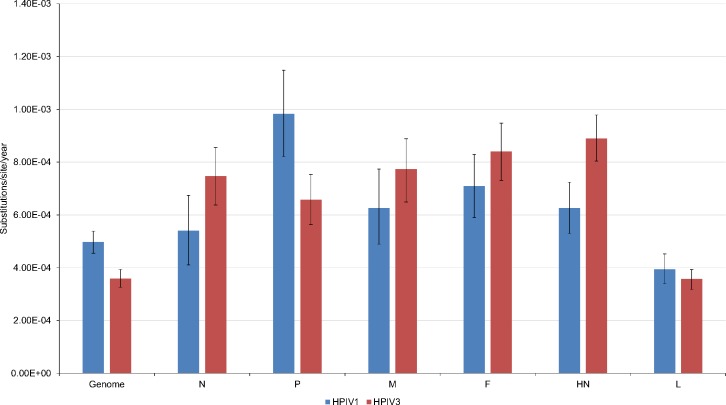
Evolutionary rates for each coding sequence in HPIV-1 and HPIV-3. Error bars represent the 95% highest posterior density.

The evolutionary rates estimated for the whole genomes were 4.97 × 10^−4^ substitutions/site/year (95% highest posterior density of 4.55 × 10^−4^ to 5.38 × 10^−4^) for HPIV-1 and 3.59 × 10^−4^ (95% highest posterior density of 3.26 × 10^−4^ to 3.94 × 10^−4^) for HPIV-3 (Fig 2). Evolutionary rates were not very different from CDS to CDS for either virus. The L gene had the lowest rate for each virus. The evolutionary rates were similar between the two viruses with HPIV-3 having a slightly lower rate overall despite having a slightly higher rate for four of the six CDS. Since these viruses have significantly different tissue tropism and epidemiology this could lead to different immunologic pressures and some differences in evolutionary rates between the two viruses. Previous studies have estimated evolutionary rates from whole genomes to be 7.61 × 10^−4^ for HPIV-1 and 4.2 × 10^−4^ for HPIV-3 and for complete HN gene sequences to be 1.12 × 10^−4^ to 1.4 × 10^−3^ for HPIV-1 and 8.39 × 10^−4^ for HPIV-3 [40–42]. The published rates are generally higher than those seen in this study, which may be due to differences in the selection of viruses used for the estimate. Regardless, the published rates are similar to those seen in this study and are typical of those seen for RNA viruses [43,44]. Another recent study found a very similar rate (3.12 × 10^−4^) for HPIV-3 when analyzing the concatenated coding regions from all genomes available in Genbank at the time [45]. Our data suggests that at least from an evolutionary rate standpoint standard vaccine approaches to HPIV-1 and 3 should be successful because the genetic and predicted antigenic change in each virus is slow enough to allow subsequent change to any vaccine with reasonable genetic monitoring of the virus population.

### HPIV-1 phylogenetic analysis

**Fig 3:**
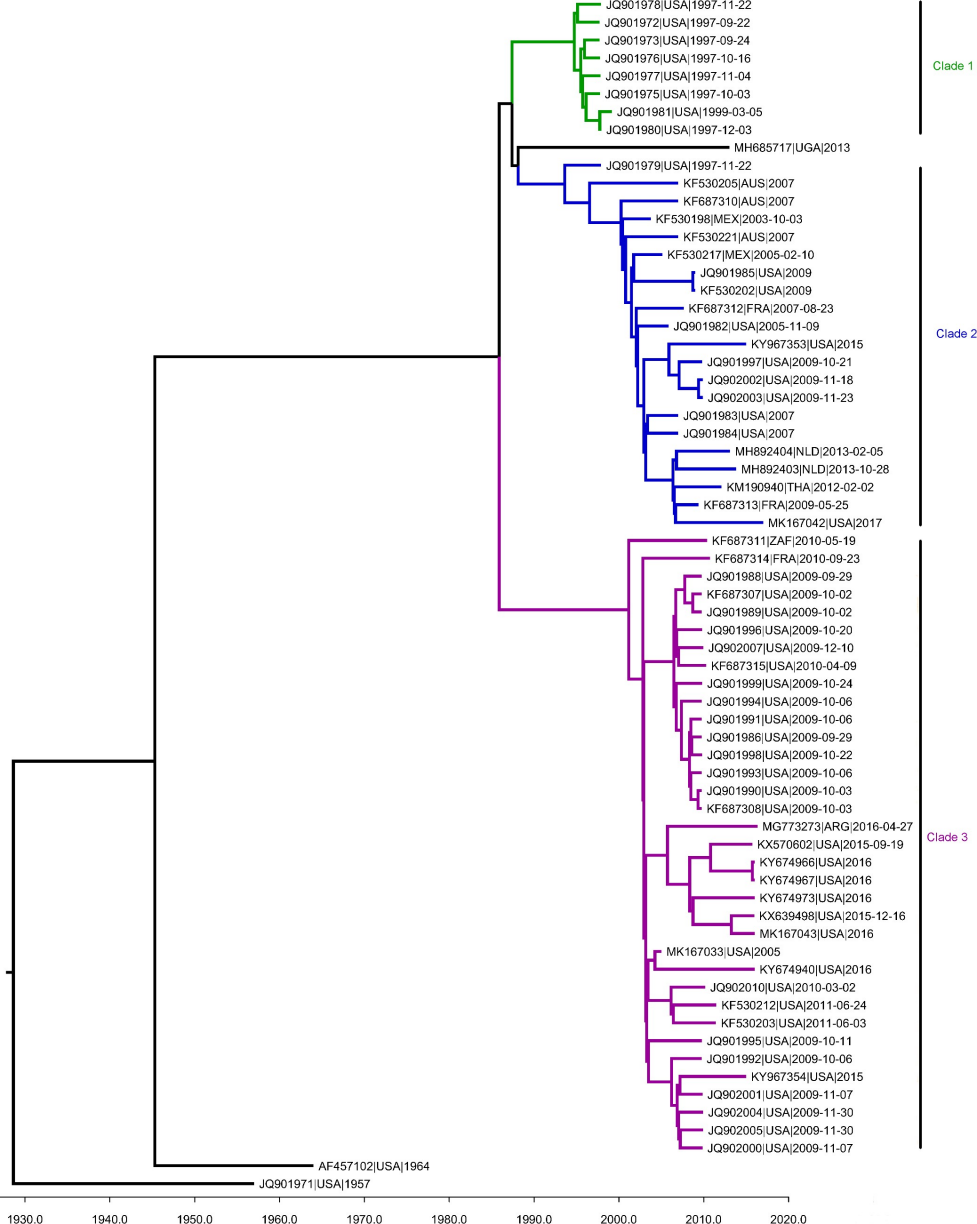
MCMC tree of HPIV-1 genome sequences. Phylogenetic tree estimated from 54 genome sequences including those sequenced in this project and those available in Genbank. The summary tree of this analysis is depicted in the figure with a time scale in years across the x-axis. The tree is divided into three clades shown with different colors and labels. Branch tips are labelled with the Genbank accession, the appropriate ISO 3166–1 alpha-3 code representing the collection country, and the year of sample collection for each sample.

The Bayesian Markov Chain Monte Carlo (MCMC) analysis of 67 HPIV-1 genome sequences identified two major HPIV-1 clades (Clades 2 and 3) circulating at the time samples were collected for this study and one clade (Clade 1) that had been circulating in the late 1990s (Fig 3). Clade 1 was very distinct with sequences from the winter of 1997 from just Wisconsin, USA, and appeared to have died off before samples were collected for our study. Clade 2 included viruses collected from 2003–2009 from Australia, France, Mexico and USA; whereas, clade 3 included viruses collected from 2009–2015, mostly from USA, with a few sequences represented from France and South Africa. Clades 2 and 3 were found to co-circulate in the USA. However, the degree of clade co-circulation in other countries was difficult to assess due to low representation of samples from other countries. However, both clades were found in France and South Africa within one or two years of each other suggesting that co-circulation may be occurring in these regions. All viruses sequenced in this study from Mexico and Australia belonged to clade 2. Since these few sequences represented all the sequences available for these two countries, it is unclear if other clades co-circulate with clade 2 in these regions. Previous studies have found these same clades co-circulating in Croatia, Japan, USA, and Vietnam [36,40,41,46,47]. Combined, the results of these studies suggest that there may have only been two main clades of HPIV-1 circulating globally during the period included in this analysis.

### HPIV-3 phylogenetic analysis

**Fig 4:**
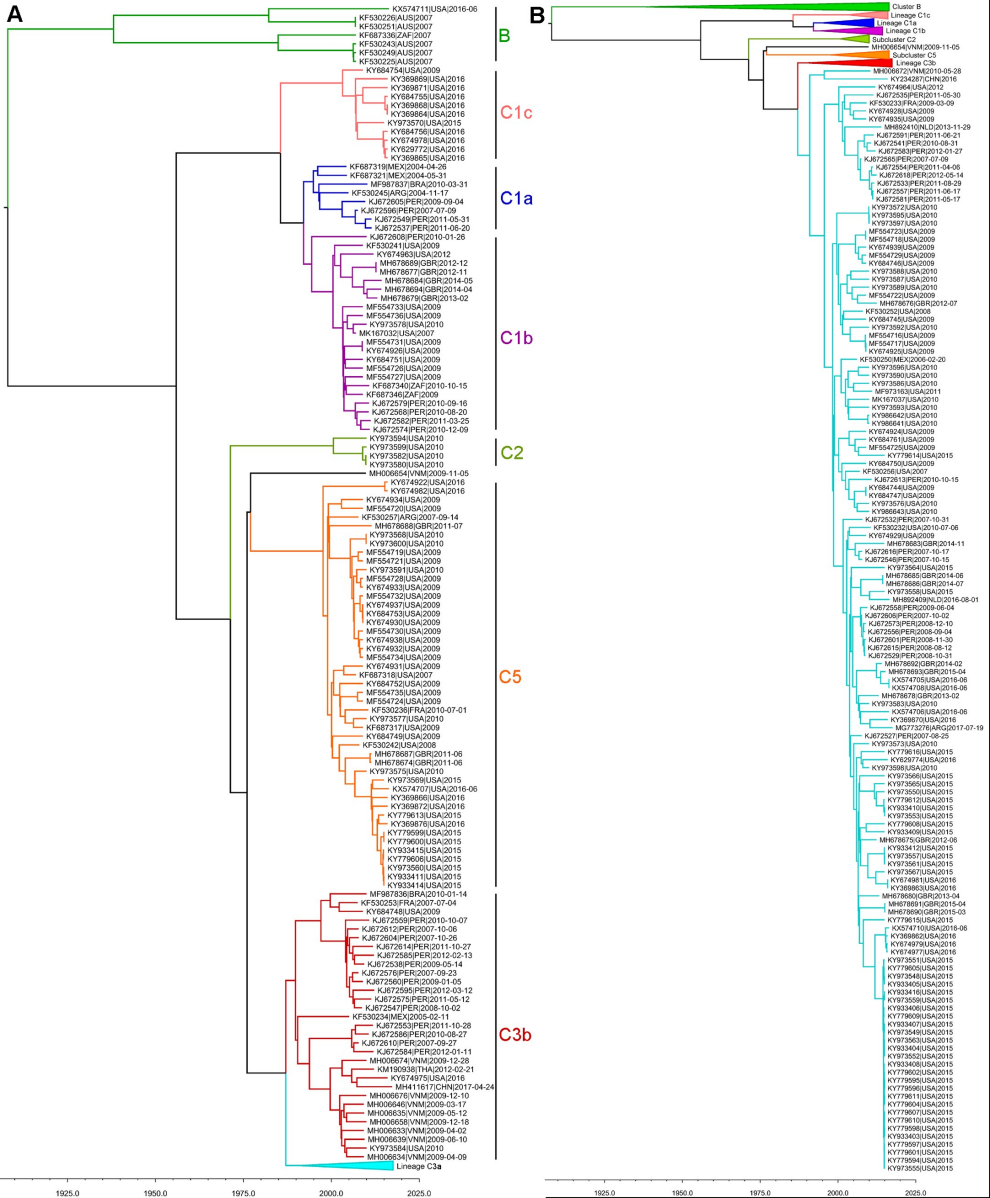
Summary MCMC tree of HPIV-3 genome sequences. Phylogenetic tree estimated from 268 genome sequences including those sequenced in this project and those available in Genbank. X-axis represents a time scale in years. Due to its size the tree has been split to make it easier to visualize. A) Lineage C3a has been collapsed. B) All subclades besides lineage C3a are collapsed. Subclades are highlighted with different colors and labels. Branch tips are labelled with the Genbank accession, the appropriate ISO 3166–1 alpha-3 code representing the collection country, and the year of sample collection for each sample.

Most HPIV-3 phylogenetic papers follow a classification system established in a study from China in 2012 [48] and further refined in 2015 [49]. In this system HPIV-3 is divided into three clusters (A, B, and C), cluster C is divided into several sub-clusters (1, 2, 3, etc.), and then some of the sub-clusters are further divided into a 3^rd^ level of classification called lineage (a, b, c, etc.). Since then some groups have split up the sub-clusters into slightly different lineages that do not completely agree between papers or added a fourth level of classification below lineage. We decided to use the more agreed upon clades mostly following what was established in Almajhdi 2015 [49]. Our MCMC analysis of 268 HPIV-3 genomes included two of the three clusters (B and C) (Fig 4). Cluster C was the largest and included viruses collected in Argentina, Brazil, China, France, Mexico, Netherlands, Peru, USA, South Africa, Thailand, United Kingdom, and Vietnam during the period 2004–2016. Our analysis included subclusters C1, C2, C3, and C5 with lineages C1a, C1b, C1c, C3a, and C3b. Subcluster C3 had the largest number of sequences with most of them belonging to lineage C3a. Cluster B was much smaller than cluster C with sequences mostly from Australia collected in 2007, one sequence from South Africa in 2007, and one recent 2016 sequence from USA. Previous studies with the HN gene have also identified a small number of sequences from cluster B in Canada and Japan [46,48,49]. Noteworthy, there were no cluster C representative sequences from Australia. Perhaps that indicates some geographical restriction of HPIV-3 transmission in and out of Australia. It would be interesting to see a larger sampling of HPIV-3 in this country over multiple years to further clarify this situation. In several countries multiple lineages of HPIV-3 were observed within the same country in a single season or year showing that multiple lineages can co-circulate. Co-circulation of multiple HPIV-3 lineages was also observed previously in a study in Croatia [46]. The co-circulation of multiple clades is not uncommon for viruses and has been observed in other paramyxoviruses like RSV subtypes A and B and human metapneumovirus with subtypes A1, A2, B1, and B2 [50,51].

## Conclusions

When we initiated this study, there were relatively few whole genome sequences available for HPIVs in GenBank. We have now added 40 HPIV-1 and 75 HPIV-3 genomes to GenBank. In the past couple of years a few other groups have also added additional HPIV genomes sequences to GenBank. Our analysis showed that multiple genetically distinct clades of both HPIV-1 and 3 circulate on a global scale with HPIV-3 having a larger number of distinct subclades that co-circulate. It may be important to consider the multiple clades of these viruses when designing vaccines in order to confer broad cross-protection. We found that some geographically distinct clades may have existed, and some appear to have died out. We further showed that HPIV-1 and 3 are evolving at similar rates and identified genomic and proteomic sites that are under positive selection. Moreover, we did not find any clear evidence that these viruses evolve by genetic recombination and demonstrate that it is possible to misinterpret dual infections as recombination events.

The whole genome HPIV sequences generated in this project will contribute to the development of robust diagnostic tools, vaccines, therapies, and a better understanding of the global and regional epidemiology and the evolution of these viruses. However, further surveillance and whole-genome sequencing is greatly needed to further understand the spatial dynamics of these important human respiratory viruses. While this and other studies have significantly increased the number of genome sequences available for HPIV-1 and 3 in GenBank, the number of genomes available for these important viruses is still very limited. Influenza A, for example, has greater than 100-fold more sequences available and it is still difficult to predict the correct vaccine strain or more prevalent subtype for any given season. Therefore, considerably more sequencing effort is needed in paramyxoviruses to further improve our knowledge of their molecular epidemiology and genetics. More studies incorporating larger numbers of sequences from other geographic regions and years are critical.

## Supporting information

S1 Text HPIV-1 Sanger sequencing primers. (TXT)

S2 Text HPIV-3 Sanger sequencing primers. (TXT)

S1 Table The sequence information of the 40 HPIV-1 genomes. (DOCX)

S2 Table The sequence information of the 75 HPIV-3 genomes. (DOCX)

S3 Table MEME episodic selection results for HPIV-1 and HPIV-3. (DOCX)

## References

[1] Parrott RH, Vargosko AJ, Kim HW, Bell JA, Chanock RM. Acute respiratory diseases of viral etiology. III. parainfluenza. Myxoviruses. Am J Public Health Nations Health. 1962;52: 907–917. 10.2105/ajph.52.6.907 14038204 PMC1523067

[2] Henrickson KJ. Parainfluenza viruses. Clin Microbiol Rev. 2003;16: 242–264. 10.1128/CMR.16.2.242-264.2003 12692097 PMC153148

[3] Henrickson KJ, Hoover S, Kehl KS, Hua W. National disease burden of respiratory viruses detected in children by polymerase chain reaction. Pediatr Infect Dis J. 2004;23: S11–8. 10.1097/01.inf.0000108188.37237.48 14730265

[4] Abedi GR, Prill MM, Langley GE, Wikswo ME, Weinberg GA, Curns AT, et al Estimates of Parainfluenza Virus-Associated Hospitalizations and Cost Among Children Aged Less Than 5 Years in the United States, 1998–2010. J Pediatr Infect Dis Soc. 2016;5: 7–13. 10.1093/jpids/piu047 26908486 PMC5813689

[5] Counihan ME, Shay DK, Holman RC, Lowther SA, Anderson LJ. Human parainfluenza virus-associated hospitalizations among children less than five years of age in the United States. Pediatr Infect Dis J. 2001;20: 646–653. 11465835 10.1097/00006454-200107000-00003

[6] Marx A, Torok TJ, Holman RC, Clarke MJ, Anderson LJ. Pediatric hospitalizations for croup (laryngotracheobronchitis): biennial increases associated with human parainfluenza virus 1 epidemics. J Infect Dis. 1997;176: 1423–1427. 10.1086/514137 9395350

[7] Bose ME, He J, Shrivastava S, Nelson MI, Bera J, Halpin RA, et al Sequencing and analysis of globally obtained human respiratory syncytial virus A and B genomes. PloS One. 2015;10: e0120098 10.1371/journal.pone.0120098 25793751 PMC4368745

[8] Noyola DE, Rodríguez-Moreno G, Sánchez-Alvarado J, Martínez-Wagner R, Ochoa-Zavala JR. Viral etiology of lower respiratory tract infections in hospitalized children in Mexico. Pediatr Infect Dis J. 2004;23: 118–123. 10.1097/01.inf.0000110269.46528.a5 14872176

[9] Noyola DE, Alpuche-Solís AG, Herrera-Díaz A, Soria-Guerra RE, Sánchez-Alvarado J, López-Revilla R. Human metapneumovirus infections in Mexico: epidemiological and clinical characteristics. J Med Microbiol. 2005;54: 969–974. 10.1099/jmm.0.46052-0 16157552

[10] Aranda-Romo S, Comas-García A, García-Sepúlveda CA, Hernández-Salinas AE, Piña-Ramírez M, Noyola DE. Effect of an immunization program on seasonal influenza hospitalizations in Mexican children. Vaccine. 2010;28: 2550–2555. 10.1016/j.vaccine.2010.01.034 20117263

[11] Gómez-Villa RJ, Comas-García A, López-Rojas V, Pérez-González LF, Sánchez-Alvarado J, Salazar-Zaragoza R, et al Effect of an infection control program on the frequency of nosocomial viral respiratory infections. Infect Control Hosp Epidemiol. 2008;29: 556–558. 10.1086/588000 18510465

[12] Weissenbacher M, Carballal G, Avila M, Salomón H, Harisiadi J, Catalano M, et al Etiologic and clinical evaluation of acute lower respiratory tract infections in young Argentinian children: an overview. Rev Infect Dis. 1990;12 Suppl 8: S889–898. 2270411 10.1093/clinids/12.supplement_8.s889

[13] Board of Science and Technology for International Development. Manual of Laboratory Procedures for Diagnosis of Respiratory Virus Infections. Washington DC: National Academy Press; 1986.

[14] Kok T, Mickan LD, Burrell CJ. Routine diagnosis of seven respiratory viruses and Mycoplasma pneumoniae by enzyme immunoassay. J Virol Methods. 1994;50: 87–100. 7714062 10.1016/0166-0934(94)90166-x

[15] Schepetiuk SK, Kok T. The use of MDCK, MEK and LLC-MK2 cell lines with enzyme immunoassay for the isolation of influenza and parainfluenza viruses from clinical specimens. J Virol Methods. 1993;42: 241–250. 8390473 10.1016/0166-0934(93)90036-q

[16] Ntshoe GM, McAnerney JM, Tempia S, Blumberg L, Moyes J, Buys A, et al Influenza epidemiology and vaccine effectiveness among patients with influenza-like illness, viral watch sentinel sites, South Africa, 2005–2009. PloS One. 2014;9: e94681–e94681. 10.1371/journal.pone.0094681 24736452 PMC3988097

[17] Lassaunière R, Kresfelder T, Venter M. A novel multiplex real-time RT-PCR assay with FRET hybridization probes for the detection and quantitation of 13 respiratory viruses. J Virol Methods. 2010;165: 254–260. 10.1016/j.jviromet.2010.02.005 20153377 PMC7112774

[18] Shih S-R, Tsao K-C, Ning H-C, Huang Y-C, Lin T-Y. Diagnosis of respiratory tract viruses in 24 h by immunofluorescent staining of shell vial cultures containing Madin-Darby Canine Kidney (MDCK) cells. J Virol Methods. 1999;81: 77–81. 10.1016/S0166-0934(99)00065-8 10488764

[19] Pretorius MA, Madhi SA, Cohen C, Naidoo D, Groome M, Moyes J, et al Respiratory Viral Coinfections Identified by a 10-Plex Real-Time Reverse-Transcription Polymerase Chain Reaction Assay in Patients Hospitalized With Severe Acute Respiratory Illness—South Africa, 2009–2010. J Infect Dis. 2012;206: S159–S165. 10.1093/infdis/jis538 23169964

[20] Cordey S, Thomas Y, Cherpillod P, Belle S van, Tapparel C, Kaiser L. Simultaneous detection of parainfluenza viruses 1 and 3 by real-time reverse transcription-polymerase chain reaction. J Virol Methods. 2009;156: 166–168. 10.1016/j.jviromet.2008.11.006 19063922 PMC7173189

[21] Li K, Shrivastava S, Brownley A, Katzel D, Bera J, Nguyen AT, et al Automated degenerate PCR primer design for high-throughput sequencing improves efficiency of viral sequencing. Virol J. 2012;9: 261-422X-9–261. 10.1186/1743-422X-9-261 23131097 PMC3548747

[22] Schobel SA, Stucker KM, Moore ML, Anderson LJ, Larkin EK, Shankar J, et al Respiratory Syncytial Virus whole-genome sequencing identifies convergent evolution of sequence duplication in the C-terminus of the G gene. Sci Rep. 2016;6 10.1038/srep26311 27212633 PMC4876326

[23] Djikeng A, Halpin R, Kuzmickas R, Depasse J, Feldblyum J, Sengamalay N, et al Viral genome sequencing by random priming methods. BMC Genomics. 2008;9: 5-2164-9–5. 10.1186/1471-2164-9-5 18179705 PMC2254600

[24] bio CLC. White paper on reference assembly in CLC assembly cell 3.0. 2010.

[25] bio CLC. White paper on de novo assembly in CLC Assembly Cell 4.0.

[26] Katoh K, Standley DM. MAFFT multiple sequence alignment software version 7: improvements in performance and usability. Mol Biol Evol. 2013;30: 772–780. 10.1093/molbev/mst010 23329690 PMC3603318

[27] Martin DP, Lemey P, Lott M, Moulton V, Posada D, Lefeuvre P. RDP3: a flexible and fast computer program for analyzing recombination. Bioinforma Oxf Engl. 2010;26: 2462–2463. 10.1093/bioinformatics/btq467 20798170 PMC2944210

[28] Delport W, Poon AF, Frost SD, Pond SLK. Datamonkey 2010: a suite of phylogenetic analysis tools for evolutionary biology. Bioinforma Oxf Engl. 2010;26: 2455–2457. 10.1093/bioinformatics/btq429 20671151 PMC2944195

[29] Pond SLK, Frost SD. Not so different after all: a comparison of methods for detecting amino acid sites under selection. Mol Biol Evol. 2005;22: 1208–1222. 10.1093/molbev/msi105 [pii] 15703242

[30] Murrell B, Wertheim JO, Moola S, Weighill T, Scheffler K, Pond SLK. Detecting individual sites subject to episodic diversifying selection. PLoS Genet. 2012;8: e1002764 10.1371/journal.pgen.1002764 22807683 PMC3395634

[31] Murrell B, Moola S, Mabona A, Weighill T, Sheward D, Pond SLK, et al FUBAR: a fast, unconstrained bayesian approximation for inferring selection. Mol Biol Evol. 2013;30: 1196–1205. 10.1093/molbev/mst030 23420840 PMC3670733

[32] Drummond AJ, Suchard MA, Xie D, Rambaut A. Bayesian phylogenetics with BEAUti and the BEAST 1.7. Mol Biol Evol. 2012;29: 1969–1973. 10.1093/molbev/mss075 22367748 PMC3408070

[33] Rambaut A, Suchard MA, Xie D, Drummond AJ. Tracer v1.6 [Internet]. 2014. Available: http://beast.bio.ed.ac.uk/Tracer

[34] Yang HT, Jiang Q, Zhou X, Bai MQ, Si HL, Wang XJ, et al Identification of a natural human serotype 3 parainfluenza virus. Virol J. 2011;8: 58 10.1186/1743-422X-8-58 21306605 PMC3045893

[35] Curran J, Pelet T, Kolakofsky D. An Acidic Activation-like Domain of the Sendai Virus P Protein Is Required for RNA Synthesis and Encapsidation. Virology. 1994;202: 875–884. 10.1006/viro.1994.1409 8030249

[36] Mizuta K, Saitoh M, Kobayashi M, Tsukagoshi H, Aoki Y, Ikeda T, et al Detailed genetic analysis of hemagglutinin-neuraminidase glycoprotein gene in human parainfluenza virus type 1 isolates from patients with acute respiratory infection between 2002 and 2009 in Yamagata prefecture, Japan. Virol J. 2011;8: 533 10.1186/1743-422X-8-533 22152158 PMC3295729

[37] Mizuta K, Tsukagoshi H, Ikeda T, Aoki Y, Abiko C, Itagaki T, et al Molecular evolution of the haemagglutinin-neuraminidase gene in human parainfluenza virus type 3 isolates from children with acute respiratory illness in Yamagata prefecture, Japan. J Med Microbiol. 2014;63: 570–577. 10.1099/jmm.0.068189-0 24464692

[38] Spriggs MK, Olmsted RA, Venkatesan S, Coligan JE, Collins PL. Fusion glycoprotein of human parainfluenza virus type 3: Nucleotide sequence of the gene, direct identification of the cleavage-activation site, and comparison with other paramyxoviruses. Virology. 1986;152: 241–251. 10.1016/0042-6822(86)90388-0 3012869

[39] Coelingh KV, Winter CC. Naturally occurring human parainfluenza type 3 viruses exhibit divergence in amino acid sequence of their fusion protein neutralization epitopes and cleavage sites. J Virol. 1990;64: 1329–1334. 1689394 10.1128/jvi.64.3.1329-1334.1990 PMC249251

[40] Beck ET, He J, Nelson MI, Bose ME, Fan J, Kumar S, et al Genome Sequencing and Phylogenetic Analysis of 39 Human Parainfluenza Virus Type 1 Strains Isolated from 1997–2010. PLOS ONE. 2012;7: e46048 10.1371/journal.pone.0046048 23029382 PMC3459887

[41] Linster M, Do LAH, Minh NNQ, Chen Y, Zhe Z, Tuan TA, et al Clinical and Molecular Epidemiology of Human Parainfluenza Viruses 1–4 in Children from Viet Nam. Sci Rep. 2018;8: 6833 10.1038/s41598-018-24767-4 29717150 PMC5931535

[42] Smielewska A, Emmott E, Ranellou K, Popay A, Goodfellow I, Jalal H. UK circulating strains of human parainfluenza 3: an amplicon based next generation sequencing method and phylogenetic analysis. Wellcome Open Res. 2018;3: 118 10.12688/wellcomeopenres.14730.2 30569021 PMC6281019

[43] Jenkins GM, Rambaut A, Pybus OG, Holmes EC. Rates of molecular evolution in RNA viruses: a quantitative phylogenetic analysis. J Mol Evol. 2002;54: 156–165. 10.1007/s00239-001-0064-3 11821909

[44] Duffy S, Shackelton LA, Holmes EC. Rates of evolutionary change in viruses: patterns and determinants. Nat Rev Genet. 2008;9: 267 10.1038/nrg2323 18319742

[45] Iketani S, Shean RC, Ferren M, Makhsous N, Aquino DB, des Georges A, et al Viral Entry Properties Required for Fitness in Humans Are Lost through Rapid Genomic Change during Viral Isolation. mBio. 2018;9 10.1128/mBio.00898-18 29970463 PMC6030562

[46] Košutić-Gulija T, Slovic A, Ljubin-Sternak S, Mlinarić-Galinović G, Forčić D. A study of genetic variability of human parainfluenza virus type 1 in Croatia, 2011–2014. J Med Microbiol. 2016;65: 793–803. 10.1099/jmm.0.000297 27302417

[47] Tsutsui R, Tsukagoshi H, Nagasawa K, Takahashi M, Matsushima Y, Ryo A, et al Genetic analyses of the fusion protein genes in human parainfluenza virus types 1 and 3 among patients with acute respiratory infections in Eastern Japan from 2011 to 2015. J Med Microbiol. 2017;66: 160–168. 10.1099/jmm.0.000431 28266286

[48] Mao N, Ji Y, Xie Z, Wang H, Wang H, An J, et al Human parainfluenza virus-associated respiratory tract infection among children and genetic analysis of HPIV-3 strains in Beijing, China. PloS One. 2012;7: e43893 10.1371/journal.pone.0043893 22937119 PMC3429441

[49] Almajhdi FN. Hemagglutinin-neuraminidase gene sequence-based reclassification of human parainfluenza virus 3 variants. Intervirology. 2015;58: 35–40. 10.1159/000369208 25592955

[50] Khor CS, Sam IC, Hooi PS, Chan YF. Displacement of predominant respiratory syncytial virus genotypes in Malaysia between 1989 and 2011. Infect Genet Evol J Mol Epidemiol Evol Genet Infect Dis. 2013;14: 357–360. 10.1016/j.meegid.2012.12.017 23305888

[51] Reiche J, Jacobsen S, Neubauer K, Hafemann S, Nitsche A, Milde J, et al Human Metapneumovirus: Insights from a Ten-Year Molecular and Epidemiological Analysis in Germany. Schildgen O, editor. PLoS ONE. 2014;9: e88342 10.1371/journal.pone.0088342 24505479 PMC3914980

