# Supplementary material for: Sequencing and analysis of globally obtained human parainfluenza viruses 1 and 3 genomes
Source: PLoS One. 2019 Jul 18;14(7):e0220057. doi: 10.1371/journal.pone.0220057 (PMC6638977; doi:10.1371/journal.pone.0220057)
Supplement: S1 Table — (DOCX) [file pone.0220057.s003.docx]

**S1 Table. The sequence information of the 40 HPIV-1 genomes.**

| **Strain Name** | **Accession** | **Sequencing Method** | **# Contigs** |
| --- | --- | --- | --- |
| HPIV1/ARG/16482/2008 | KF530206 | Illumina, Ion Torrent | 1 |
| HPIV1/AUS/53/2007 | KF530221 | Sanger, Illumina, Ion Torrent | 1 |
| HPIV1/AUS/54/2007 | KF687310 | Sanger | 1 |
| HPIV1/AUS/57/2007 | KF530205 | Sanger, Illumina, Ion Torrent, 454 | 1 |
| HPIV1/CHE/1109273044/2011 | KF530222 | Illumina, Ion Torrent | 2* |
| HPIV1/CHE/1110180075/2011 | KF530200 | Illumina, Ion Torrent | 3* |
| HPIV1/FRA/26503037/2006 | KF530208 | Illumina, Ion Torrent | 1* |
| HPIV1/FRA/27343049/2007 | KF530215 | Illumina, Ion Torrent | 3* |
| HPIV1/FRA/27344044/2007 | KF687312 | Sanger | 1 |
| HPIV1/FRA/29171082/2009 | KF530201 | Illumina, Ion Torrent | 1 |
| HPIV1/FRA/29212070/2009 | KF530223 | Illumina, Ion Torrent | 2* |
| HPIV1/FRA/29221106/2009 | KF687313 | Sanger | 1 |
| HPIV1/FRA/29342077/2009 | KF530197 | Illumina, Ion Torrent | 1 |
| HPIV1/FRA/30384043/2010 | KF687314 | Sanger | 1* |
| HPIV1/FRA/30393061/2010 | KF530218 | Illumina, Ion Torrent | 2* |
| HPIV1/FRA/31026021/2011 | KF530219 | Illumina, Ion Torrent | 3* |
| HPIV1/FRA/31202043/2011 | KF530210 | Illumina, Ion Torrent | 3* |
| HPIV1/FRA/31211174/2011 | KF530224 | Illumina, Ion Torrent | 4* |
| HPIV1/FRA/31225147/2011 | KF530209 | Illumina, Ion Torrent | 2* |
| HPIV1/MEX/447/2003 | KF687309 | Sanger | 4* |
| HPIV1/MEX/482/2003 | KF530207 | Illumina, Ion Torrent | 2* |
| HPIV1/MEX/495/2003 | KF530198 | Sanger, Illumina, 454 | 1* |
| HPIV1/MEX/1521/2005 | KF530217 | Sanger, Illumina, Ion Torrent | 1 |
| HPIV1/MEX/1842/2005 | KF530220 | Illumina, Ion Torrent | 2* |
| HPIV1/MEX/3040/2006 | KF530196 | Illumina, Ion Torrent | 1 |
| HPIV1/USA/32193A/2010 | KF687315 | Sanger | 1 |
| HPIV1/USA/33095J/2011 | KF530211 | Illumina, Ion Torrent | 1 |
| HPIV1/USA/35686C/2011 | KF530213 | Illumina, Ion Torrent | 4* |
| HPIV1/USA/35745H/2011 | KF687316 | Sanger | 2* |
| HPIV1/USA/38078A/2011 | KF530203 | Sanger, Illumina, Ion Torrent | 1 |
| HPIV1/USA/38081A/2011 | KF530212 | Sanger, Illumina, Ion Torrent | 1 |
| HPIV1/USA/38761A/2011 | KF530216 | Illumina, Ion Torrent | 2* |
| HPIV1/USA/629-2/2009 | KF530202 | Illumina, Ion Torrent, 454 | 1 |
| HPIV1/USA/629-D00712/2009 | KF687307 | Sanger | 1 |
| HPIV1/USA/629-D02161/2009 | KF687308 | Sanger | 1 |
| HPIV1/ZAF/879/2010 | KF687311 | Sanger | 1 |
| HPIV1/ZAF/2530/2008 | KF530204 | Illumina, Ion Torrent | 2* |
| HPIV1/ZAF/2614/2010 | KF530214 | Illumina, Ion Torrent | 2* |
| HPIV1/ZAF/2754/2010 | KF530195 | Illumina, Ion Torrent | 3* |
| HPIV1/ZAF/3267/2010 | KF530199 | Illumina, Ion Torrent | 2* |

* indicates that the genome finishing status is draft and contains sequencing gaps due to either low-coverage or low sequence quality in some areas of the genome.
