# Supplementary material for: Sequencing and analysis of globally obtained human parainfluenza viruses 1 and 3 genomes
Source: PLoS One. 2019 Jul 18;14(7):e0220057. doi: 10.1371/journal.pone.0220057 (PMC6638977; doi:10.1371/journal.pone.0220057)
Supplement: S2 Table — (DOCX) [file pone.0220057.s004.docx]

**S2 Table. The sequence information of the 75 HPIV-3 genomes.**

| **Strain Name** | **Accession** | **Sequencing Method** | **# Contigs** |
| --- | --- | --- | --- |
| HPIV3/AUS/1/2007 | KF530226 | Illumina, Ion Torrent | 1 |
| HPIV3/AUS/2/2007 | KF530237 | Illumina, Ion Torrent | 2* |
| HPIV3/AUS/3/2007 | KF530243 | Illumina, 454 | 1 |
| HPIV3/AUS/4/2007 | KF530238 | Illumina, Ion Torrent | 3* |
| HPIV3/AUS/5/2007 | KF530251 | Illumina, 454 | 1 |
| HPIV3/AUS/6/2007 | KF530249 | Illumina, 454 | 1 |
| HPIV3/AUS/7/2007 | KF530230 | Illumina, 454 | 1 |
| HPIV3/AUS/8/2007 | KF530235 | Illumina, Ion Torrent | 3* |
| HPIV3/AUS/9/2007 | KF530225 | Illumina, Ion Torrent | 1 |
| HPIV3/ARG/10068/2004 | KF530245 | Illumina, 454 | 1 |
| HPIV3/ARG/12894/2006 | KF687341 | Sanger | 5* |
| HPIV3/ARG/13009/2006 | KF687343 | Sanger | 1 |
| HPIV3/ARG/14555/2007 | KF687342 | Sanger | 2* |
| HPIV3/ARG/15318/2007 | KF530257 | Illumina, 454 | 1 |
| HPIV3/CHE/1103040115/2011 | KF530246 | Illumina, Ion Torrent | 1* |
| HPIV3/CHE/1104190065/2011 | KF530254 | Illumina, Ion Torrent | 3* |
| HPIV3/CHE/1105230137/2011 | KF687358 | Sanger | 1 |
| HPIV3/FRA/26232106/2006 | KF687349 | Sanger | 1 |
| HPIV3/FRA/27045070/2007 | KF687350 | Sanger | 3* |
| HPIV3/FRA/27273076/2007 | KF530253 | Illumina, Ion Torrent | 1 |
| HPIV3/FRA/28015134/2008 | KF687351 | Sanger | 2* |
| HPIV3/FRA/29111069/2009 | KF530233 | Illumina, Ion Torrent | 1 |
| HPIV3/FRA/29122056/2009 | KF687352 | Sanger | 1* |
| HPIV3/FRA/29144018/2009 | KF530227 | Illumina, Ion Torrent | 3* |
| HPIV3/FRA/30254042/2010 | KF687353 | Sanger | 1* |
| HPIV3/FRA/30261045/2010 | KF687354 | Sanger | 1* |
| HPIV3/FRA/30264021/2010 | KF530236 | Illumina, Ion Torrent | 1 |
| HPIV3/FRA/31065118/2011 | KF687356 | Sanger | 2* |
| HPIV3/FRA/31084077/2011 | KF687355 | Sanger | 4* |
| HPIV3/MEX/1077/2004 | KF687319 | Sanger | 1* |
| HPIV3/MEX/1099/2004 | KF687320 | Sanger | 1 |
| HPIV3/MEX/1110/2004 | KF687321 | Sanger | 1* |
| HPIV3/MEX/1512/2005 | KF687322 | Sanger | 1* |
| HPIV3/MEX/1527/2005 | KF687323 | Sanger | 1 |
| HPIV3/MEX/1526/2005 | KF530234 | Illumina, Ion Torrent | 1 |
| HPIV3/MEX/2545/2006 | KF530250 | Illumina, Ion Torrent | 1 |
| HPIV3/MEX/2822/2006 | KF687324 | Sanger | 1 |
| HPIV3/MEX/2831/2006 | KF687325 | Sanger | 1* |
| HPIV3/MEX/2841/2006 | KF687326 | Sanger | 1* |
| HPIV3/MEX/3047/2006 | KF530248 | Illumina, Ion Torrent | 3* |
| HPIV3/MEX/3593/2007 | KF687327 | Sanger | 1 |
| HPIV3/MEX/3640/2007 | KF687328 | Sanger | 1 |
| HPIV3/MEX/4159/2008 | KF687329 | Sanger | 2* |
| HPIV3/MEX/4169/2008 | KF687330 | Sanger | 1* |
| HPIV3/MEX/4601/2009 | KF687331 | Sanger | 3* |
| HPIV3/MEX/4638/2009 | KF687332 | Sanger | 1 |
| HPIV3/USA/629-1/2006 | KF687347 | Sanger | 1 |
| HPIV3/USA/629-2/2006 | KF530239 | Illumina, Ion Torrent | 2* |
| HPIV3/USA/629-10/2009 | KF530229 | Illumina, Ion Torrent | 1 |
| HPIV3/USA/629-10/2009 | KF687317 | Sanger | 1 |
| HPIV3/USA/629-D1/2009 | KF530241 | Illumina, Ion Torrent | 1 |
| HPIV3/USA/8745D/2011 | KF687348 | Sanger | 1* |
| HPIV3/USA/10991B/2010 | KF530232 | Illumina, Ion Torrent | 1 |
| HPIV3/USA/35084A/2011 | KF687357 | Sanger | 1 |
| HPIV3/USA/629-D00155/2007 | KF530231 | NGS | 2* |
| HPIV3/USA/629-D00687/2008 | KF530242 | NGS | 1 |
| HPIV3/USA/629-D01363/2008 | KF530252 | NGS | 1 |
| HPIV3/USA/629-D01547/2007 | KF530255 | NGS | 2* |
| HPIV3/USA/629-D01929/2007 | KF530256 | Illumina, Ion Torrent | 1 |
| HPIV3/USA/629-D01959/2007 | KF687318 | Sanger | 1 |
| HPIV3/USA/629-D02180/2007 | KF530240 | Illumina, Ion Torrent | 2* |
| HPIV3/USA/629-D02313/2006 | KF530247 | Illumina, Ion Torrent | 1 |
| HPIV3/ZAF/2262/2008 | KF530244 | Illumina, Ion Torrent | 5* |
| HPIV3/ZAF/2516/2008 | KF687344 | Sanger | 1 |
| HPIV3/ZAF/3066/2008 | KF687337 | Sanger | 3* |
| HPIV3/ZAF/3227/2010 | KF687338 | Sanger | 4* |
| HPIV3/ZAF/3238/2010 | KF687339 | Sanger | 6* |
| HPIV3/ZAF/3314/2010 | KF530228 | Illumina, Ion Torrent | 3* |
| HPIV3/ZAF/3372/2010 | KF687340 | Sanger | 1 |
| HPIV3/ZAF/6281/2009 | KF687345 | Sanger | 1* |
| HPIV3/ZAF/6538/2009 | KF687346 | Sanger | 1 |
| HPIV3/ZAF/1145978/2007 | KF687336 | Sanger | 1 |
| HPIV3/ZAF/1181009/2007 | KF687333 | Sanger | 1 |
| HPIV3/ZAF/1183071/2007 | KF687335 | Sanger | 3* |
| HPIV3/ZAF/1200334/2007 | KF687334 | Sanger | 1* |

* indicates that the genome finishing status is draft and contains sequencing gaps due to either low-coverage or low sequence quality in some areas of the genome.
