# Supplementary material for: Sequencing and analysis of globally obtained human parainfluenza viruses 1 and 3 genomes
Source: PLoS One. 2019 Jul 18;14(7):e0220057. doi: 10.1371/journal.pone.0220057 (PMC6638977; doi:10.1371/journal.pone.0220057)
Supplement: S3 Table — (DOCX) [file pone.0220057.s005.docx]

S3 Table. MEME Episodic Selection Results for HPIV-1 and HPIV-3.

| Virus | Gene | Codon | SLAC p-value | FEL p-value | FUBAR Posterior Probability | MEME p-value |
| --- | --- | --- | --- | --- | --- | --- |
| 1 | N | 197 | 0.371 | 0.247 | 0.918 | **<0.001** |
| 1 | P | 106 | 0.204 | 0.070 | 0.877 | **0.047** |
| 1 | P | 136 | 0.653 | 0.650 | 0.543 | **0.001** |
| 1 | P | 137 | 0.584 | 0.344 | 0.776 | **0.022** |
| 1 | P | 159 | 0.162 | **0.029** | **0.980** | **<0.001** |
| 1 | P | 349 | 0.504 | 0.208 | 0.727 | **0.024** |
| 1 | P | 493 | 0.741 | 0.443 | 0.229 | **0.002** |
| 1 | M | 312 | 0.198 | 0.093 | 0.946 | **0.011** |
| 1 | F | 5 | 0.230 | **0.036** | **0.973** | **0.045** |
| 1 | F | 555 | 0.376 | 0.155 | 0.887 | **0.020** |
| 1 | HN | 355 | 0.398 | 0.127 | 0.841 | **0.050** |
| 1 | L | 26 | 0.388 | 0.225 | 0.867 | **0.001** |
| 1 | L | 444 | 0.445 | 0.167 | 0.69 | **<0.001** |
| 1 | L | 448 | 0.862 | 0.454 | 0.186 | **0.006** |
| 1 | L | 1153 | 0.444 | 0.388 | 0.551 | **0.031** |
| 1 | L | 1541 | 0.468 | 0.189 | 0.910 | **<0.001** |
| 1 | L | 1720 | 0.347 | 0.061 | 0.938 | **0.049** |
| 1 | L | 1733 | 0.532 | 0.322 | 0.678 | **<0.001** |
| 1 | L | 1735 | 0.403 | 0.635 | 0.714 | **0.012** |
| 1 | L | 2121 | 0.511 | 0.485 | 0.243 | **<0.001** |
| 1 | L | 2170 | 0.667 | 0.288 | 0.503 | **0.047** |
| 3 | N | 440 | **0.005** | **0.002** | **1.000** | **0.004** |
| 3 | P | 104 | 0.132 | **0.027** | 0.939 | **0.038** |
| 3 | P | 109 | **0.026** | **0.008** | **0.961** | **0.014** |
| 3 | P | 142 | 0.098 | **0.010** | **0.955** | **0.017** |
| 3 | P | 178 | 0.105 | **0.032** | 0.938 | **0.047** |
| 3 | P | 196 | 0.097 | **0.013** | **0.962** | **0.021** |
| 3 | P | 302 | 0.156 | **0.012** | **0.964** | **0.002** |
| 3 | F | 108 | **0.024** | **0.032** | **0.982** | **0.048** |
| 3 | F | 517 | 0.764 | 0.572 | 0.496 | **0.012** |
| 3 | HN | 21 | 0.749 | 0.295 | 0.079 | **0.028** |
| 3 | HN | 58 | **0.034** | **0.019** | **0.974** | **0.039** |
| 3 | HN | 208 | 0.234 | 0.201 | 0.849 | **0.018** |
| 3 | L | 166 | 0.102 | 0.099 | 0.871 | **0.048** |
| 3 | L | 275 | 0.504 | 0.304 | 0.580 | **0.002** |
